# Nonaneurysmal abdominal aortitis in an 82-year-old woman presenting with pyrexia and back pain: a case report

**DOI:** 10.4076/1752-1947-3-8958

**Published:** 2009-09-09

**Authors:** Manoj Kumar, Tariq Barakat, Grace Timmons, Ahmed Mudawi

**Affiliations:** 1 Department of Vascular Surgery, Queen Elizabeth Hospital, Gateshead, Tyne and Wear, UK

## Abstract

**Introduction:**

Infective aortitis has become uncommon since the advent of antibiotic therapy. Aortitis, presenting as a localised perforation in a non-aneurysmal aorta, is extremely rare. We report the case of an 82-year-old woman who was diagnosed with localised perforation of a non-aneurysmal abdominal aorta secondary to staphylococcus aortitis.

**Case presentation:**

An 82-year-old woman presented with a history of a sudden onset of back pain and pyrexia. A clinical examination did not reveal any significant findings attributable to her sepsis. As her clinical condition deteriorated rapidly, adequate resuscitation was commenced. Appropriate serology and radiological investigations, including a computed tomography scan, were performed. The computed tomography scan revealed a diagnosis of a non-aneurysmal infective abdominal aortitis with evidence of localised perforation. This was successfully treated under local anaesthetic with endovascular aortic repair and appropriate antibiotics. She recovered fully and was completely asymptomatic a year later.

**Conclusion:**

A detailed assessment is essential in the diagnosis of this condition as it can frequently be missed on initial evaluation of the affected patient. Clinical features are often nonspecific and can include fever, leucocytosis and bacteremia in the absence of a pulsatile or expansile mass. The patient may also complain of back pain, as in this case report. Thorough assessment, timely investigation and endovascular intervention prevented a potentially fatal condition in our patient.

## Introduction

Infective aortitis has become uncommon since the advent of antibiotic therapy [1]. Aortitis presenting as a localised perforation in a non-aneurysmal aorta is even more unusual. We report the case of an 82-year-old woman presenting with a history of lower back pain and pyrexia. She was diagnosed with abdominal aortitis secondary to *Staphylococcus aureus*, which was complicated by an aortic perforation. The aortitis was successfully treated under local anaesthetic by endovascular repair and appropriate antibiotics.

## Case presentation

An 82-year-old woman presented with a 2-week history of a sudden onset of acute lower back pain and worsening mobility. She did not report any other significant medical history. A clinical examination revealed a patient who was clinically dehydrated. She was pyrexial (39°C), tachycardic (pulse rate of 105/minute) and hypotensive (blood pressure of 90/50 mmHg on admission). Abdominal, chest and musculoskeletal examinations and urine analysis did not reveal any significant findings that could be attributed to her worsening sepsis. She had a leucocytosis level of 19,600, which increased to 40,700 in 24 hours, accompanied by worsening pain and pyrexia. She was also noted to be oliguric. Her lower back pain worsened and resulted in her becoming bedridden within 24 hours.

**Figure 1:**
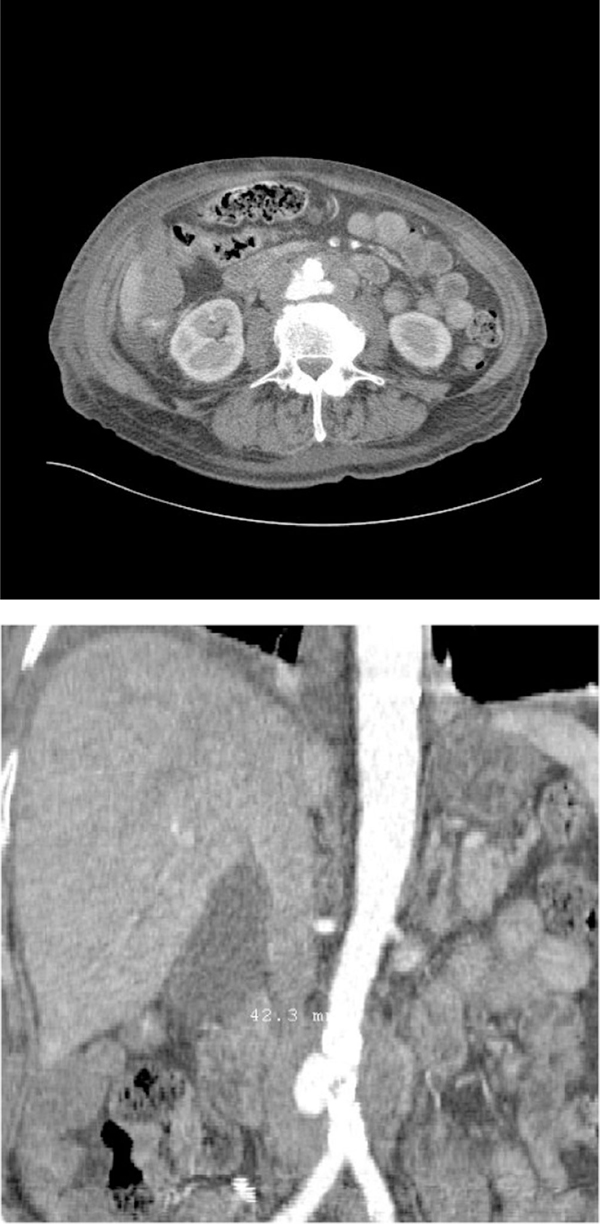
****(A)** A computed tomography scan showing localised perforation of infrarenal aorta and stranding of surrounding tissue**. **(B)** Two-dimensional coronal reconstruction of lower abdomen demonstrating position and size of leak.

Appropriate resuscitative measures were initiated upon admission. She was also commenced on broad spectrum intravenous antibiotics. An ultrasound scan and a computed tomography (CT) scan of her abdomen were performed. These revealed an abnormal infrarenal aorta with evidence of stranding of periaortic fat planes and a localised perforation resulting in a contained leak into the surrounding soft tissue (Figures 1A and 1B). The iliacs were not involved and there was no evidence of an aneurysm. The blood cultures that were taken on admission confirmed the presence of *Staphylococcal aureus*. The overall findings were consistent with abdominal aortitis. A white cell scan was not performed as this would have caused a delay to the operation on this patient. She was classified as high-risk for a general or spinal anaesthetic, therefore an endovascular aortic repair (EVAR) was carried out under local anaesthetic. Her common femoral artery was exposed and punctured under direct vision and a sheath was introduced. A 20-mm tube stent graft (Zenith® Endovascular Graft Leg Extension) was then cantered over the site of perforation in the infrarenal aorta. The distal landing zone was proximal to the bifurcation. A check angiography was performed pre and post deployment. This confirmed the correct positioning of the stent and the exclusion of the leak.

After the procedure, our patient had a full complement of pulses in both lower limbs and minimal pain control was required. Added risks and complications of a general or spinal anaesthetic were avoided. Antibiotics (Linezolid ®) of 600 mg IV twice daily were started after the operation and continued for approximately 3 weeks. The antibiotics were selected following positive blood cultures and sensitivities.

**Figure 2:**
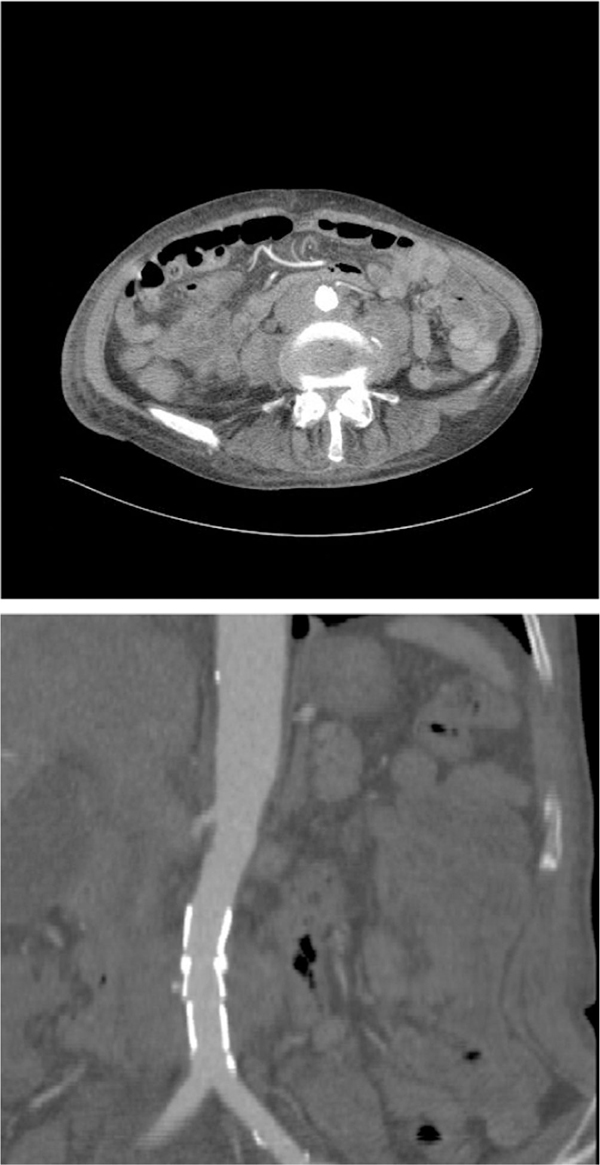
****(A)** Effective seal of localised perforation by stent graft**. **(B)** Two-dimensional coronal reconstruction of lower abdomen demonstrating effective seal of localised perforation of infrarenal aorta.

**Figure 3:**
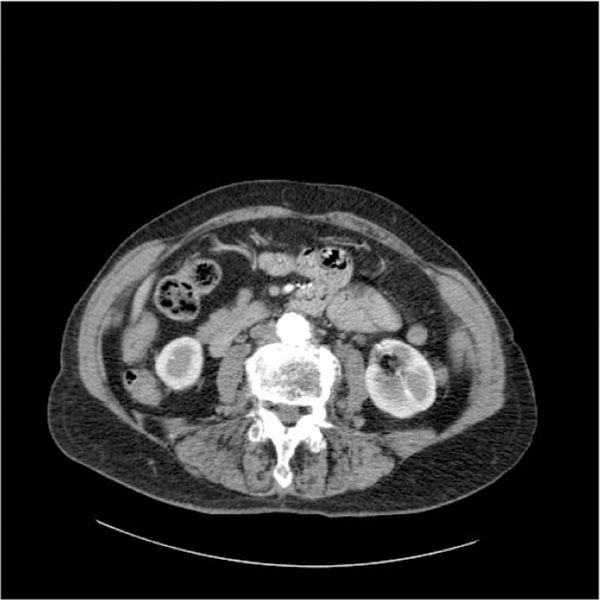
**A computed tomography scan angiogram 1 year after endovascular aortic repair showing no evidence of aortitis or leak**.

Our patient's problem with sepsis was resolved and her overall clinical condition continued to improve with the signs of inflammation returning to normal levels. A CT scan (Figures 2A and 2B) performed 2 weeks after the operation revealed that the endoluminal stent had effectively sealed the aortic leak. The patient was asymptomatic and well at a follow-up check-up one year later. A CT scan (Figure 3) revealed no evidence of aortitis or leak.

## Discussion

Aortitis is a rare condition involving inflammation of the aorta and it can cause aortic dilatation, fibrous thickening, osteal stenosis or dissection, resulting in aortic insufficiency or rupture. Inflammatory aneurysm of the abdominal aorta (IAAA) affects approximately 3% to 10% of the population diagnosed with an abdominal aortic aneurysm [2].

The principal aetiological agents of the disorder include *Salmonella*, *Staphylococcus aureus*, and *Escherichia coli*, although in some cases the aetiology of the disorder remains unclear. It has been suggested that both autoimmune and genetic depositions may also contribute to the disorder [2].

Aortitis presenting as a perforation is an unusual entity. A literature search revealed only one other case reported by Stephens *et al*., which was treated by open repair [3]. A detailed assessment is required in the diagnosis of this condition as it is known to be easily missed on initial evaluation of the affected patient [3]. Clinical features are often nonspecific and can include fever, leucocytosis and bacteremia in the absence of a pulsatile or expansile mass and, in cases where dissection is present, the patient may complain of back pain, as in this case report. Gomes et al. reported positive blood cultures in 70% and a palpable abdominal mass in 53% of cases of infected aneurysms [4]. However, even when suspicion is high, many patients do not present with such clinical signs or symptoms.

Treatment of aortitis is by a combination of antibiotic therapy and aortic replacement. Three reports described patients who were treated with antibiotics only and all three patients died [5]-[7]. Traditionally, treatment of infectious aortitis was done by open repair [3]; however, reports from the Eurostar database [8] and a meta-analysis of EVAR of inflammatory aortic aneurysms [9] have all concluded that operative and midterm results are as effective and comparable to standard open abdominal aortic aneurysm repair.

## Conclusion

Most reports of endovascular treatment of inflammatory aneurysms have been performed under general anaesthetic or spinal anaesthetic. Treatment options for this patient were limited because of the high operative risk under a general or spinal anaesthetic.

A thorough assessment and early radiological investigations ensured a timely diagnosis and appropriate intervention in this case. Nonspecific clinical features of this condition can result in a delay in diagnosis with a potential fatal outcome.

## Abbreviations

CT: computed tomography; EVAR: endovascular aortic repair; IAAA: inflammatory aneurysm of the abdominal aorta.

## Consent

Written informed consent was obtained from the patient for publication of this case report. A copy of the written consent is available for review by the Editor-in-Chief of this journal.

## Competing interests

The authors declare that they have no competing interests.
